# Effect of GnRH 7 Days Before Presynchronization With Simultaneous PGF_2α_ and GnRH on Reproductive Outcomes in Holstein Dairy Cows

**DOI:** 10.3389/fvets.2020.574516

**Published:** 2020-10-22

**Authors:** Andrew M. Hubner, Phillip M. G. Peixoto, Joshua Hillesheim, Igor F. Canisso, Fabio S. Lima

**Affiliations:** ^1^Department of Veterinary Clinical Medicine, College of Veterinary Medicine, University of Illinois, Urbana, IL, United States; ^2^Department of Comparative Biosciences, College of Veterinary Medicine, University of Illinois, Urbana, IL, United States; ^3^Lena Veterinary Clinic, Lena, IL, United States

**Keywords:** presynchronization, timed Ai, anovular, ovulation, pregnancy per AI

## Abstract

We evaluated if an additional GnRH injection 7 days before pre-synchronization with simultaneous PGF_2α_ and GnRH (PG+G) would improve responses to presynchronization, synchronization, and pregnancy per AI (P/AI). We hypothesized that administering GnRH 7 days before PG+G would increase ovulation and corpus luteum (CL) presence at the PG+G, improve response to OvSynch treatments and P/AI. Holstein cows were blocked by parity and randomly assigned to either a PG+G (Control, *n* = 205); or to GnRH followed 7 days later by PG+G (ExtG, *n* = 201). At enrollment, Control was left untreated, whereas ExtG received GnRH. Seven days after enrollment, Control and ExtG received PG+G followed by OvSynch 7 days later (GnRH, 7 days PGF_2α_, 56 h GnRH, 16 h timed AI). Ovarian dynamics were assessed using ultrasonography in a subset of cows (*n* = 53 for Control; and *n* = 50 for ExtG) at each treatment, except the 2^nd^ GnRH of OvSynch. Pregnancy diagnosed at 32- and 67-days post AI. Ovulation at enrollment tended (*P* = 0.06) to be higher for ExtG, but ovulation was not different at PG+G (*P* = 0.41) and first GnRH of the OvSynch (*P* = 0.25). There was a tendency (*P* = 0.08) for ExtG to have larger CL than Control at PGF_2α_ of the OvSynch. There were no differences in CL and follicle sizes in any other treatment point assessed. There were no differences (*P* = 0.12) in luteolysis between treatments after PG+G. Overall P/AI was similar between treatments on Day 32 (Control = 33.0% vs. ExtG = 34.6%, *P* = 0.75) and 67 (Control = 31.8% vs. ExtG = 32.5%, *P* = 0.29) post AI. There was a tendency for an interaction between treatment and parity (*P* = 0.09) for P/AI at day 67 post-AI. In multiparous cows, ExtG tended to have greater P/AI than Control, whereas, in primiparous cows Control tended to have greater P/AI than ExtG at day 67 post-AI. In conclusion, the effects of GnRH 7 days before PG+G presynchronization lead to positive and negative tendencies, respectively, in multiparous and primiparous cows for P/AI at day 67 post-AI and needs further investigation.

## Introduction

There is a body of evidence indicating that 25 to 30% of lactating dairy cows remain anovular (defined by the absence of a functional CL) at 50 to 70 days in milk (DIM) (1–6). Anovular cows at the beginning of estrous synchronization programs such as OvSynch are remarkably less fertile than cows with a functional CL, with differences in pregnancies/AI (P/AI) varying from 12 to 23% between cyclic and anovular cows (4, 6, 7). Throughout the years, a series of pre-synchronization programs involving GnRH were developed to help overcome the reproductive performance issues in the anovular cows (8–10). Double-OvSynch demonstrated improved outcomes when compared to the classic Presynch (two PGF_2α_ treatments 14 days apart) followed by OvSynch 12 days later (11).

Pre-synchronization programs containing GnRH have improved pregnancy outcomes in anovular cows (8, 9). However, many farms and veterinarians in charge of performing treatments during herd check still opt for programs such as the classic Presynch, followed by the OvSynch 14 days apart, where fewer days per week for treatments are required (12). A presynchronization that helps anovular cows such as the Double-OvSynch, with an additional injection of PGF_2α_ at the end of the program for improved luteolysis, requires 4 days per week for treatments (13). The G6G presynchronization (PGF_2α_ followed 2 days later by GnRH and 6 days later first GnRH of the OvSynch) has fewer overall treatments than the Double-OvSynch, but still requires treatments 4 days per week to complete all of the injections when combined with OvSynch (8). A recent presynchronization program attempted to resolve the issues of having a presynchronization that is helpful for anovular cows but also reduces the number of days per week on which treatments are administered (10). The presynchronization program was constituted of simultaneous GnRH and PGF_2α_ (PG+G) 7 days before the OvSynch (10). The PG+G program reduced the number of days on which treatments are administered to two rather than 4 days. The P/AI for this program were similar to the G6G program (PG+G = 47% vs. G6G = 54%), albeit authors reported a 35% probability for type-II error due to a limited number of cows (10). Moreover, there was a suboptimal response at the time of PG+G, with only 58% of cows ovulating in response to the GnRH of presynchronization, and only 66% of the cows that had complete luteolysis in response to PGF_2α_ (10). The suboptimal responses lead to a 15% decrease in cows with a functional CL at the beginning of the OvSynch (91 vs. 76%). Additionally, the percentage of cows with a new estrous cycle following presynchronization was 24% lower than cows receiving the G6G at (69 vs. 45%) (10). Although no differences in ovulation to the first GnRH of OvSynch were reported in this study (10), the 68% reported was lower than the 80% of 85% reported for presynchronization programs with GnRH such as G6G and Double-OvSynch (8, 14, 15). Previous work has shown that ovulation to GnRH injection is more likely to occur if a new follicular wave began 5–9 days earlier (16) and cows that ovulate to the first GnRH of OvSynch have better pregnancy per AI (8, 9, 17, 18). Complete regression of a CL only occurs with a single exogenous treatment of PGF_2α_ after day 5 of the estrous cycle (19, 20). Thus, possible improvements of the presynchronization program with GnRH and PGF_2α_ may be possible by reducing the randomness in regard to which day of the estrous cycle cows receive these treatments. Any additional steps to improve the PG+G presynchronization may only be attractive if it does not increase the number of days per week on which treatments are administered.

The objective of this study was to determine if additional treatment of GnRH 7 days before PG+G would improve responses to the PG+G program. We hypothesized that the additional treatment with GnRH 7 days before PG+G would increase the number of cows having a responsive ovulatory follicle and corpus luteum at the pre-synchronization. The increased number of cows with responsive ovulatory follicles and CL at PG+G would translate to better responses to OvSynch treatments and higher P/AI without increasing the number of treatment days per week necessary to complete the program.

## Materials and Methods

### Cows, Housing, and Diets

The present study was carried out from August 2018 to November 2019 on a commercial dairy farm in northern Illinois, milking ~400 cows. The cows were milked three times per day, having a rolling herd average milk production of 13,397 kg per year with a 3.6% milk fat and a 3.1% milk protein. Cows were housed in freestall barns and fed a total mixed ration (TMR) once daily, having free access to feed and water. The TMR consisted of corn silage, alfalfa hay, and corn and soybean meal-based concentrate formulated to meet or exceed nutrient recommendations for lactating dairy cows at the previously mentioned production level based on the Nutrient Requirements of Dairy Cattle, 2001 (21). All animal procedures followed the recommendations of the Guide for the Care and Use of Agricultural Animals in Agricultural Research and Teaching (FASS, 2010; accessed at https://www.adsa.org/Portals/_default/SiteContent/docs/AgGuide3rd/Chapter07.pdf).

### Study Design

**Figure 1:**
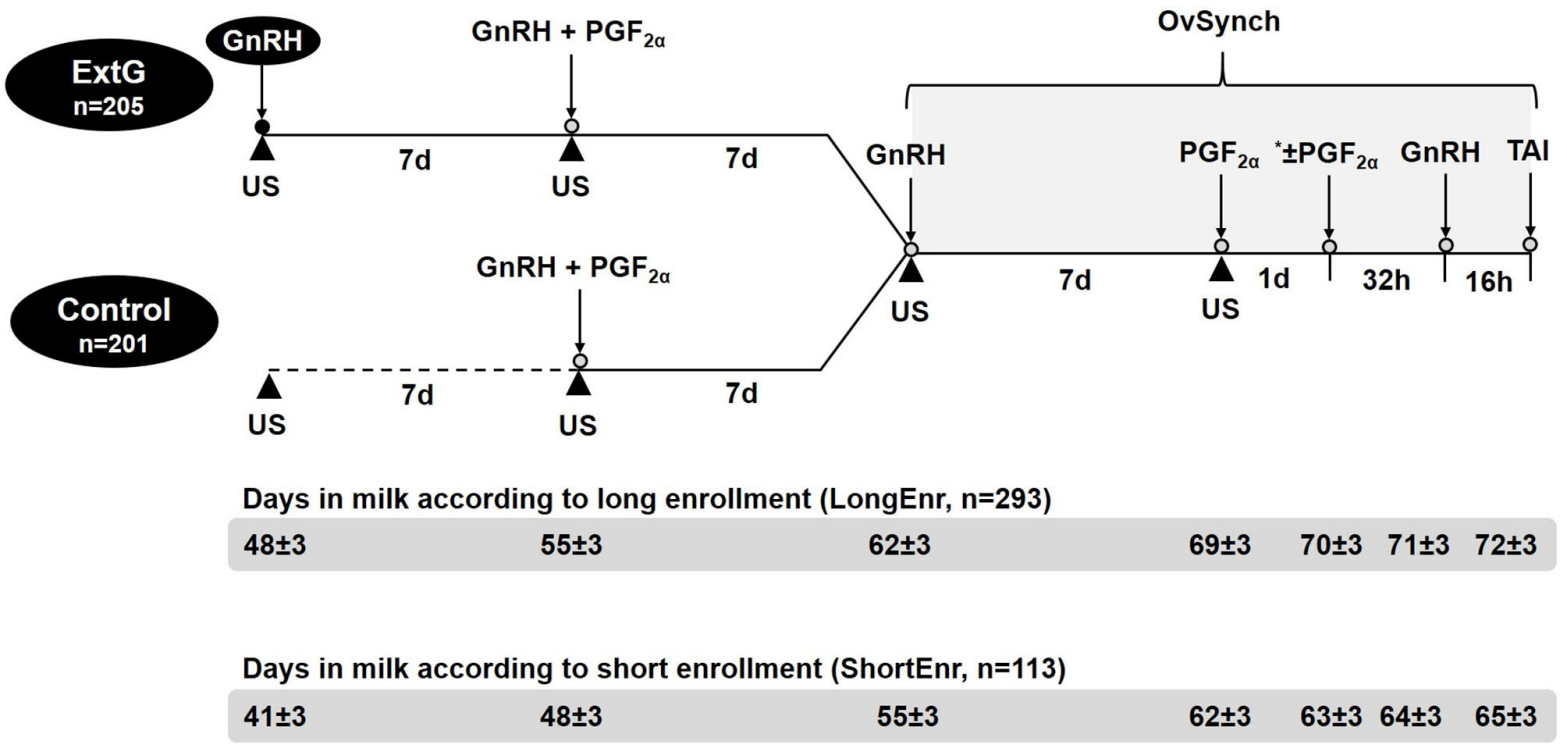
Schematic illustration of the timeline for presynchronization and synchronization treatments and ultrasonography examination (US) to assess follicular and luteal dynamics. *A subset of cows enrolled in both treatments in the study (*n* = 118 for Control; and *n* = 115 for ExtG) received a second PGF_2α_ 24 h after the first PGF_2α_ of the OvSynch.

Weekly cohorts of lactating Holstein dairy cows at 48 ± 3 DIM (LongEnr, *n* = 293), then part-way through the study at 41 ± 3 DIM (ShortEnr, *n* = 113) were blocked by parity and randomly assigned to either a simultaneous GnRH and PGF_2α_ presynchronization (Control, *n* = 205); or to GnRH followed 7 days later by simultaneous GnRH and PGF_2α_ presynchronization (ExtG, *n* = 201). Cows in the Control and ExtG groups received, respectively, no treatment or GnRH (1 ml containing 100 μg of gonadorelin acetate per ml, Gonabreed, Parnell, Overland Park, KS) at 48 ± 3 DIM. Seven days later, at 55 ± 3 DIM, cows from both Control and ExtG received simultaneous treatment of GnRH and PGF_2α_ (2 ml of cloprostenol sodium-containing 250 μg per ml, Estroplan, Parnell, Overland Park, KS). Seven days later at 62 ± 3 DIM, cows from the Control and ExtG groups were started on OvSynch (22, 23) receiving GnRH treatment, followed 7 d later (at 69 ± 3 DIM) by PGF_2α_ treatment followed ~56 h later by a second treatment with GnRH and timed AI at ~16 h as depicted in Figure 1. A second PGF_2α_ was administered at 24 h following the PGF_2α_ of OvSynch for a subset of cows enrolled in both treatments of the study (*n* = 118 for Control; and *n* = 115 for ExtG). These were the final 233 cows enrolled in the study and were administered an additional PGF2α in an effort to improve pregnancy outcomes. Treatments on the day of the start of OvSynch (Additional GnRH for ExtG group, GnRH, and PGF_2α_ of presynchronization, first GnRH of OvSynch, and PGF_2α_ of the OvSynch) were administered by the herd veterinarian. Injections on the other days (Second GnRH of OvSynch and then part way through the study, second PGF_2α_) were given by the farm personnel. Treatments of PGF_2α_ and GnRH were administered with multi-dose syringes using 18-gauge 1.5-inch needles in the semimembranosus or semitendinosus muscles of the cows. One experienced technician working for an AI company performed all AI for the duration of the study, except for 2 weeks that were performed by another experienced technician from the same AI company. Both technicians were blinded to the treatments. Commercial semen from multiple sires purchased by the farm was used.

### Ovarian Ultrasonography, Follicular and Luteal Responses, and Pregnancy Diagnosis

A subset of cows (*n* = 53 for control; and *n* = 50 for ExtG) had ovaries mapped during the study to characterize the physiological responses to exogenous hormone treatments. Transrectal ultrasound was performed on the day enrollment (day of extra GnRH in ExtG group), day of simultaneous GnRH and PGF_2α_, the day the first GnRH of OvSynch, and day of PGF_2α_ of the OvSynch. All ultrasonographic examinations were carried out using an Easi-Scan coupled with a 7.5 MHz linear probe (Easi-Scan, BCF Technologies, Bellshill, United Kingdom). The largest diameter (D) of each follicle >9 mm and CL >16 mm were estimated using gridlines on the ultrasound image (24). Fluid-filled cavities in CL were measured in the same fashion. The area and volume of the CL were calculated by subtracting the cavity area or volume from the total CL area or volume. The area was calculated using π *x* 0.5*D*, and volume was calculated using 4/3 *x π x* 0.5*D*. Ovulation was characterized by the appearance of a new CL in the place of a previous ovulatory follicle on either ovary. Luteolysis was characterized by the disappearance of a CL after treatment with PGF_2α_. Pregnancy diagnosis was performed by the herd veterinarian at 32 days post timed-AI and then rechecked 67 days post timed-AI. The presence of an amniotic vesicle containing an embryo with a heartbeat was used as the criterion to determine pregnancy. Pregnancy loss was calculated as the number of cows that lost a pregnancy between d 32 and 67 after AI divided by the number of cows diagnosed pregnant on d 32 after AI.

### Statistical Analyses

Power analyses were performed to calculate the sample size using G Power 3 (Universität Düsseldorf, Germany) (25, 26). Sample sizes were calculated to detect a difference in cows having ovulation to the first GnRH and P/AI for the first service. The expected percent of ovulation to the first GnRH of the OvSynch was 65% for the Control and 85% for the ExtG group, based on a previous study using PG+G presynchronization (10), and expected improvement with extra GnRH. Considering the difference of 20% between treatments, an α error probability of 5%, power β (the probability of a type II error) of 80%, and a one-tailed test, a minimum of 42 experimental units per treatment were deemed necessary to assess a difference in ovulation to the first GnRH of the OvSynch. Under the assumptions of improved synchronization responses, we surmised that P/AI would increase by 13 percentage points. Considering the difference of 13 percentage points between treatments, an α error probability of 5%, power β (the probability of a type II error) of 80%, and a one-tailed test, a minimum of 178 experimental units per treatment were deemed necessary to assess differences in P/AI. Because of potential attrition, ~10% more cows were added to both treatments.

Descriptive data were analyzed using the FREQ procedure of SAS version 9.4 (SAS/STAT; SAS Institute Inc., Cary, NC). Categorical data (presence of CL, presence of ovulatory follicle, ovulation, luteolysis, P/AI) were analyzed by logistic regression using the LOGISTIC procedure of SAS fitting a binary distribution. Backward stepwise logistic regression models were used, and variables were continuously removed from the models by the Wald statistic criterion when *P* > 0.10. The continuous data (size of follicle and CL volume) were analyzed using the GLIMMIX procedure of SAS with models fitting a Gaussian distribution. Data were tested for normality of residuals, and non-normally distributed data were transformed before analysis if improvement in residual distribution was observed. The models tested included the effect of treatment and the covariates parity, season, and the interactions of treatment by parity and treatment by season. The covariance structure that resulted in the smallest Akaike's information criterion was selected for the model. Differences with *P* ≤ 0.05 were considered significant, and those with 0.05 < *P* ≤ 0.10 were considered tendencies.

## Results

### Follicular Dynamics

**Figure 2:**
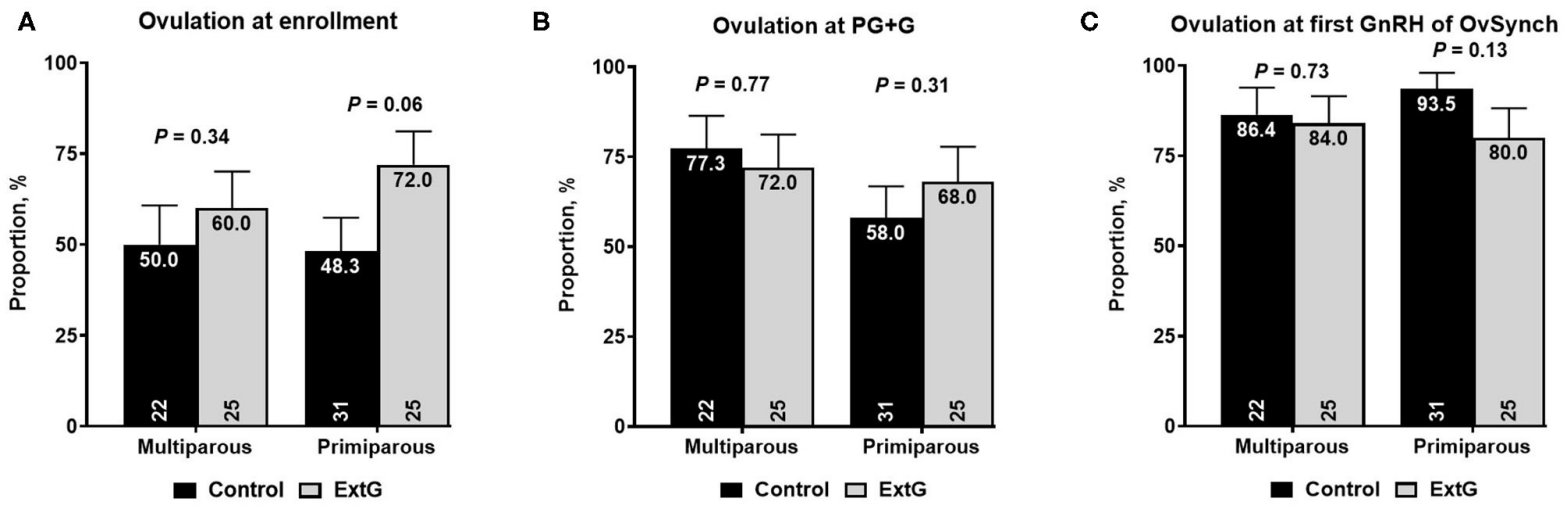
Bar graph illustrating the proportion of cows ovulating **(A)** at enrollment, **(B)** at simultaneous PGF_2α_ and GnRH (PG+G), and **(C)** at first GnRH of OvSynch according to treatment (Control vs. ExtG) in multiparous and primiparous cows. The proportion is listed on the top, whereas the number of cows per treatment is at the bottom. Error bars represent the standard error of the mean.

Amongst follicular responses, there was no difference in the percent of cows with an ovulatory follicle at enrollment (*P* = 0.65), at PG+G (*P* = 0.12), at first GnRH of Ovsynch (*P* = 0.34), and PGF_2α_ of the OvSynch (*P* = 0.29) between Control and ExtG (Table 1). The size of the ovulatory follicle at enrollment (*P* = 0.70), at PG+G (*P* = 0.30), at first GnRH of Ovsynch (*P* = 0.44), and at PGF_2α_ of the OvSynch (*P* = 0.35) was not different between treatments (Table 1). Cows receiving the extra GnRH 7 days before the PG+G tended to have greater ovulation at the time of GnRH (*P* = 0.06) when compared with the Control cows not receiving the extra dose of GnRH (Table 1). However, there was no difference in ovulation at the PG+G (*P* = 0.41) or the first GnRH treatment of the OvSynch (*P* = 0.25) between Control and ExtG cows (Table 1). There were effects of parity and interaction of treatment by parity observed for ovulation at enrollment, thus models were run for the effects of treatment in each parity separately. In primiparous cows, a tendency (*P* = 0.06) for increased ovulation at enrollment in ExtG when compared to Control was present. In contrast, in multiparous, no differences (*P* = 0.34) in ovulation at enrollment between ExtG and Control was found (Figure 2A). Ovulation at PG+G was not different between ExtG and Control in primiparous (*P* = 0.31) and multiparous (*P* = 0.77) cows (Figure 2B). Ovulation at first GnRH of OvSynch was not different between ExtG and Control in primiparous (*P* = 0.13) and multiparous (*P* = 0.73) cows (Figure 2C).

**Table 1:** Follicular dynamics responses at the time of enrollment (Extra GnRH treatment for ExtG group), time of presynchronization with simultaneous GnRH and PGF_2α_, treatments for cows enrolled Control and ExtG presynchronization programs.

	Treatments^*^		***P*-value**
	**Control**	**ExtG**	
**Item**			
Cows with ovulatory follicle at enrollment, % (*n*/*n*)	83.0 (44/53)	84.0 (42/50)	0.65
Cows with ovulatory follicle at GnRH and PGF_2α_ presynchronization, % (*n*/*n*)	96.2 (51/53)	88.0 (44/50)	0.12
Cows with ovulatory follicle at the first GnRH of OvSynch, % (*n*/*n*)	90.6 (48/53)	86.0 (43/50)	0.34
Cows with ovulatory follicle at the PGF_2α_ of OvSynch, % (*n*/*n*)	86.8 (46/53)	92.0 (46/50)	0.29
Size of the ovulatory follicle at enrollment, mm ± SEM	19.5 ± 1.5	18.8 ± 0.8	0.70
Size of the ovulatory follicle at GnRH and PGF_2α_ presynchronization, mm ± SEM	18.9 ± 1.1	17.3 ± 1.1	0.30
Size of the ovulatory follicle at the first GnRH of OvSynch, mm ± SEM	19.2 ± 1.1	17.9 ± 1.2	0.44
Size of the ovulatory follicle at the PGF_2α_ of OvSynch, mm ± SEM	17.6 ± 1.0	16.5 ± 1.1	0.35
Ovulation at enrollment to GnRH (ExtG) or spontaneous ovulation (Control), % (*n*/*n*)	49.1 (26/53)	66.0 (33/50)	0.06
Ovulation at GnRH and concurrent PGF_2α_ for presynchronization, % (*n*/*n*)	66.0 (35/53)	70.0 (35/50)	0.41
Ovulation at the first GnRH of OvSynch, % (*n*/*n*)	90.6 (48/53)	82.0 (41/50)	0.25

* *Cows were randomly allocated to two either Control or ExtG treatments. The control treatment consisted of presynchronization with GnRH and PGF_2α_, followed by OvSynch 7 days later, while ExtG treatment consisted of a treatment of GnRH followed simultaneous GnRH and PGF_2α_ 7 days later followed by OvSynch after 7 days*.

### Luteal Dynamics

**Figure 3:**
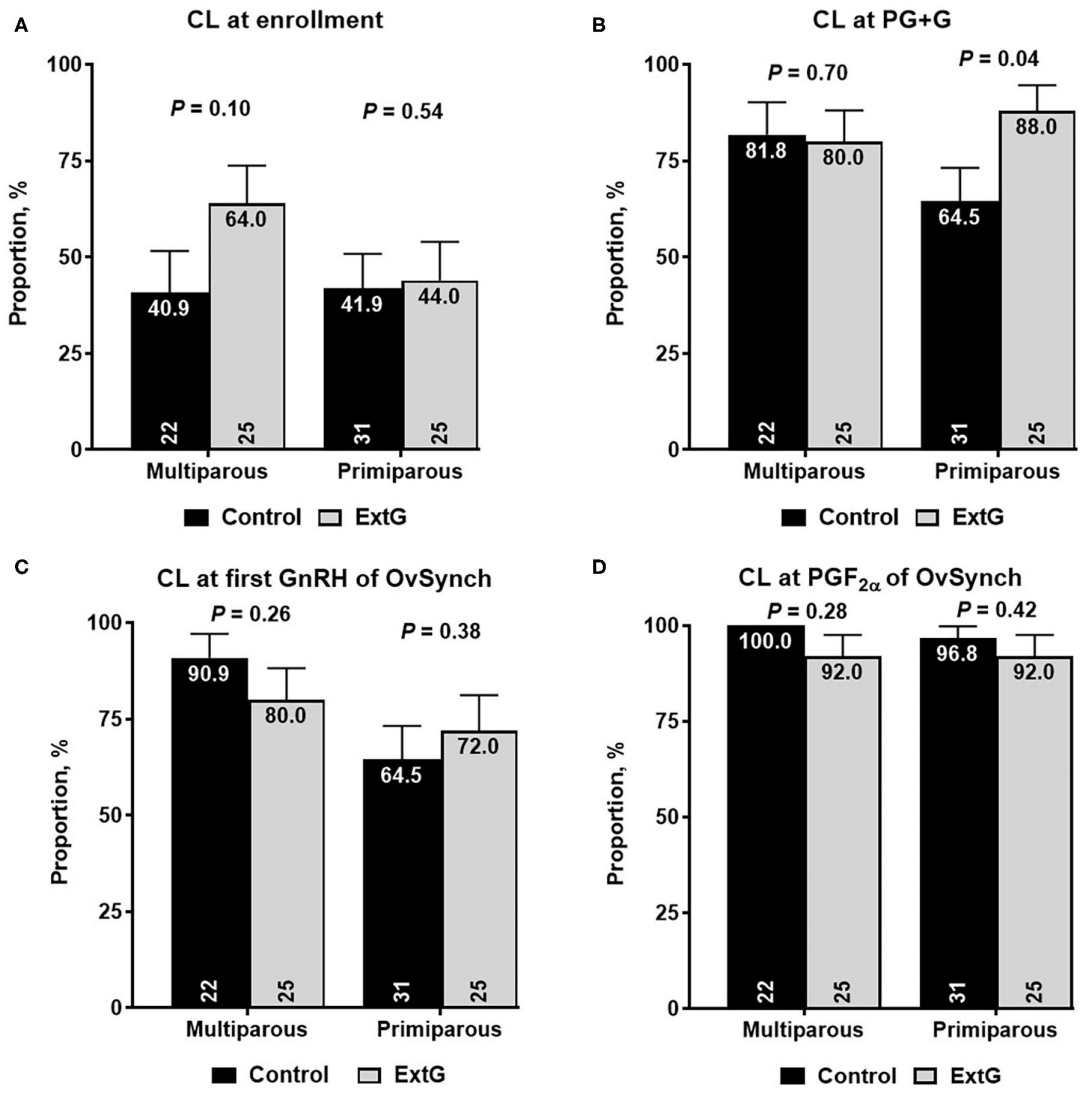
Bar graph illustrating the proportion of cows with corpus luteum (CL) **(A)** at enrollment, **(B)** at simultaneous PGF2α and GnRH (PG+G), **(C)** at first GnRH of OvSynch, and **(D)** at PGF2α of OvSynch according to treatment (Control vs. ExtG) in multiparous and primiparous cows. The proportion is listed on the top, whereas the number of cows per treatment is at the bottom. Error bars represent the standard error of the mean.

Amongst luteal responses, the proportion of cows with a CL at enrollment (*P* = 0.14), at PG+G (*P* = 0.11), at first GnRH of Ovsynch (*P* = 0.95), and at PGF_2α_ of the OvSynch (*P* = 0.14) did not differ between Control and ExtG cows (Table 2). The total CL volume tended to be larger for ExtG cows than for Control cows at PGF_2α_ of the OvSynch (*P* = 0.08). However, no differences between treatments for total CL volume at enrollment (*P* = 0.96), at simultaneous GnRH and PGF_2α_ treatment (*P* = 0.49), or at first GnRH of Ovsynch (*P* = 0.81) were detected in the study (Table 2). There was no difference in luteolysis to the PGF_2α_ given concurrently with GnRH (*P* = 0.42) for Control and ExtG cows (Table 2). An effect of parity for CL at first GnRH of OvSynch was present with multiparous cows having a greater (85.1 ± 5.2 vs. 68.8 ± 6.3, *P* = 0.04) presence of CL than primiparous. Effects of parity for CL presence at enrollment and PG+G and interaction of treatment by parity were observed. Thus, models were run for the effects of treatment in each parity separately. For the presence of CL at enrollment, no effect of treatment for primiparous (*P* = 0.54) and multiparous (*P* = 0.10) cows were present (Figure 3A). For primiparous cows CL presence at PG+G was greater for ExtG than Control (*P* = 0.04), but in multiparous cows, no difference (*P* = 0.70) for the presence of CL at PG+G was found (Figure 3B). The presence of CL at first GnRH of OvSynch was not different between ExtG and Control in primiparous (*P* = 0.38) and multiparous (*P* = 0.26) cows (Figure 3C). Likewise, CL presence at PGF_2α_ of the OvSynch was not different between ExtG and Control in primiparous (*P* = 0.42) and multiparous (*P* = 0.28) cows (Figure 3D).

**Table 2:** Luteal dynamics responses at the time of enrollment (Extra GnRH treatment for ExtG group), time of presynchronization with simultaneous GnRH and PGF_2α_, treatments for cows enrolled Control and ExtG presynchronization programs.

	Treatments^*^		***P*-value**
	**Control**	**ExtG**	
**Item**			
Cows with a corpus luteum (CL) at enrollment, % (*n*/*n*)	41.5 (22/53)	54.0 (27/50)	0.14
Cows with a CL at GnRH and PGF_2α_ presynchronization, % (*n*/*n*)	71.7 (38/53)	82.0 (41/50)	0.11
Cows with CL at the first GnRH of OvSynch, % (*n*/*n*)	75.5 (40/53)	76.0 (38/50)	0.95
Cows with CL at the PGF_2α_ of OvSynch, % (*n*/*n*)	98.1 (52/53)	92.0 (46/50)	0.14
CL volume at enrollment, mm^3^ ± SEM	9627 ± 1075	9550 ± 971	0.96
CL volume at GnRH and PGF_2α_ presynchronization, mm^3^ ± SEM	8950 ± 798	9717 ± 783	0.49
CL volume at the first GnRH of OvSynch, mm^3^ ± SEM	8000 ± 846	8288 ± 868	0.81
CL volume at the PGF_2α_ of OvSynch, mm^3^ ± SEM	9401 ± 811	11405 ± 820	0.08
Luteolysis to the PGF_2α_ given concurrent with GnRH, % (*n*/*n*)	89.5 (34/38)	95.1 (39/41)	0.42

* *Cows were randomly allocated to two either Control or ExtG treatments. The control treatment consisted of presynchronization with GnRH and PGF_2α_, followed by OvSynch 7 days later, while ExtG treatment consisted of treatment of GnRH followed 7 days later by simultaneous GnRH and PGF_2α_ followed by OvSynch after 7 days*.

### Pregnancy Outcomes

**Figure 4:**
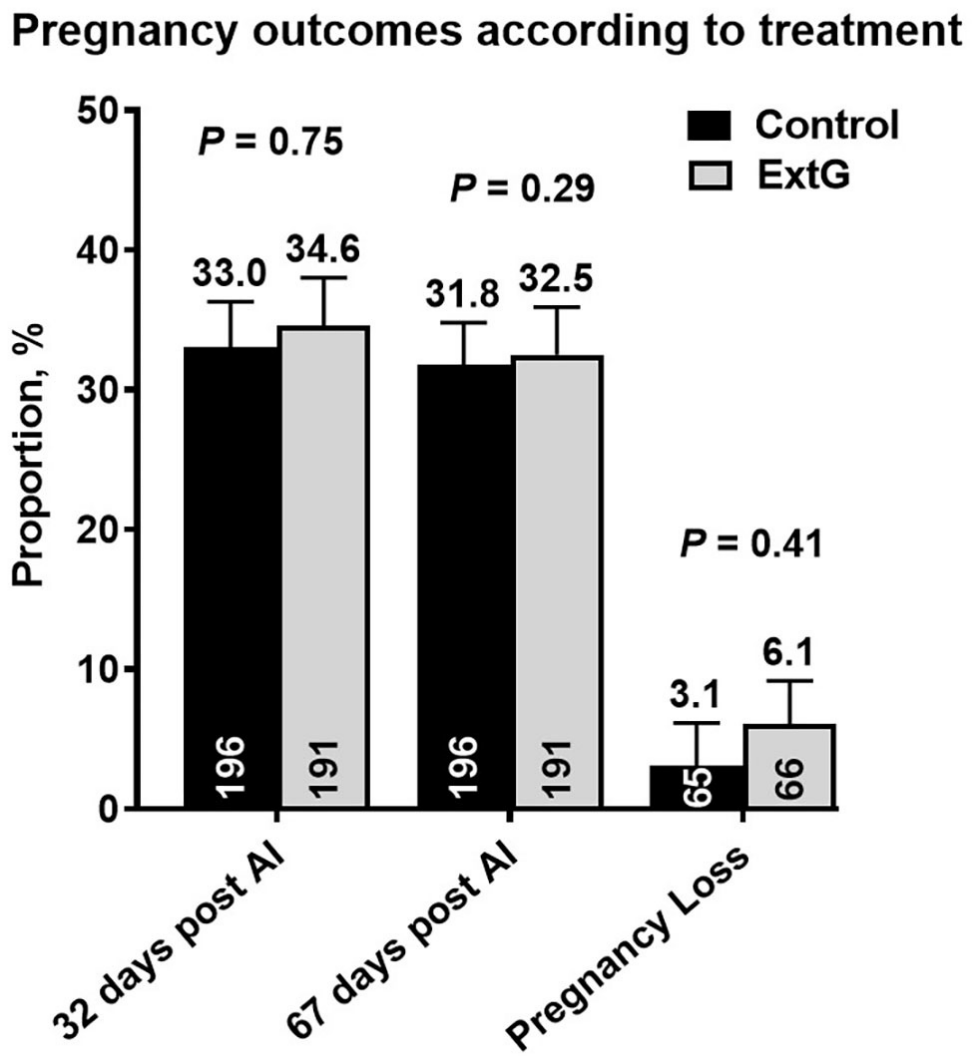
Bar graph illustrating the proportion of pregnant cows at days 32 and 67 post-AI and proportion of pregnancy loss according to the effects of treatment (Control vs. ExtG). The mean proportion for each bar is listed above each error bar, whereas the number of cows per treatment is at the bottom of each bar. Error bars represent the standard error of the mean.

**Figure 5:**
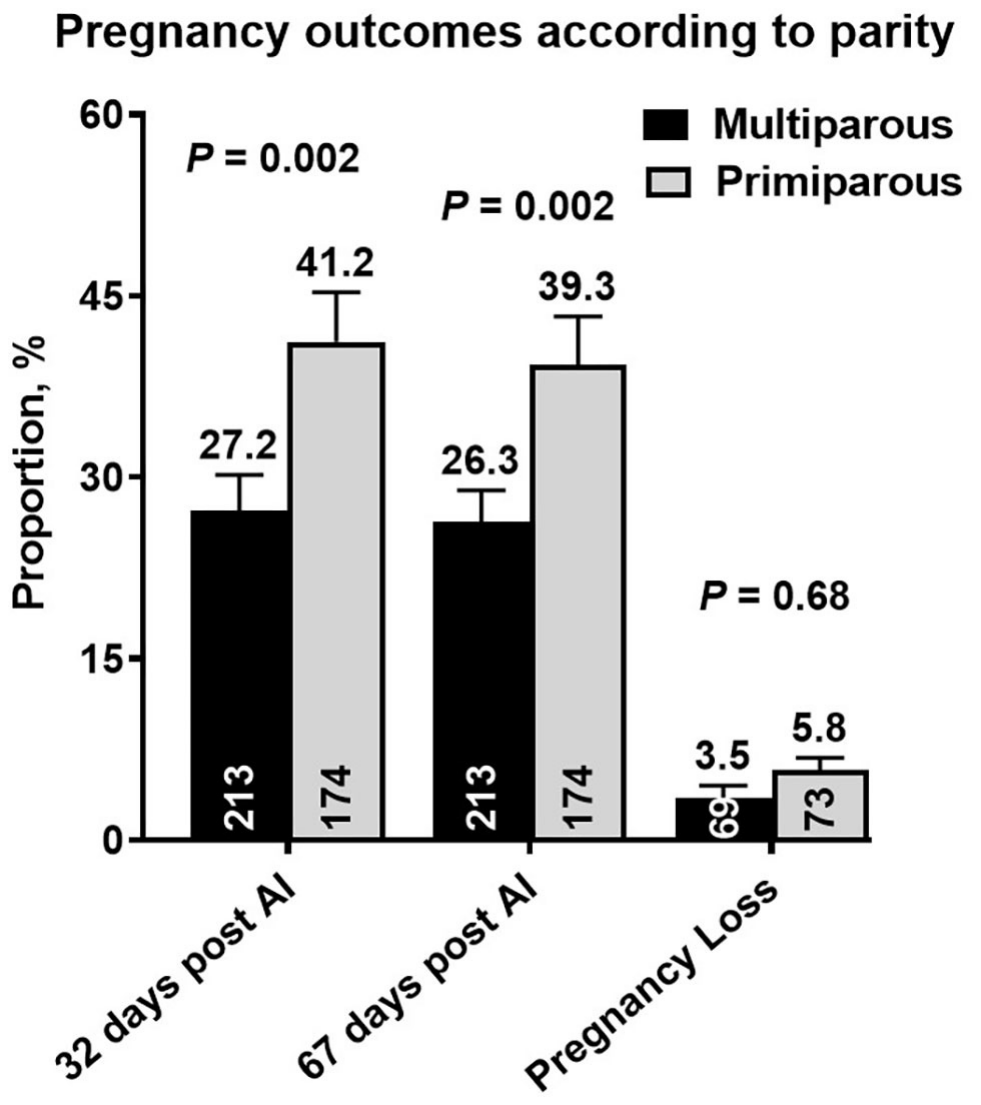
Bar graph illustrating the proportion of pregnant cows at days 32 and 67 post-AI and proportion of pregnancy loss according to the effects of parity (Multiparous vs. Primiparous). The mean proportion for each bar is listed above each error bar, whereas the number of cows per treatment is at the bottom of each bar. Error bars represent the standard error of the mean.

**Figure 6:**
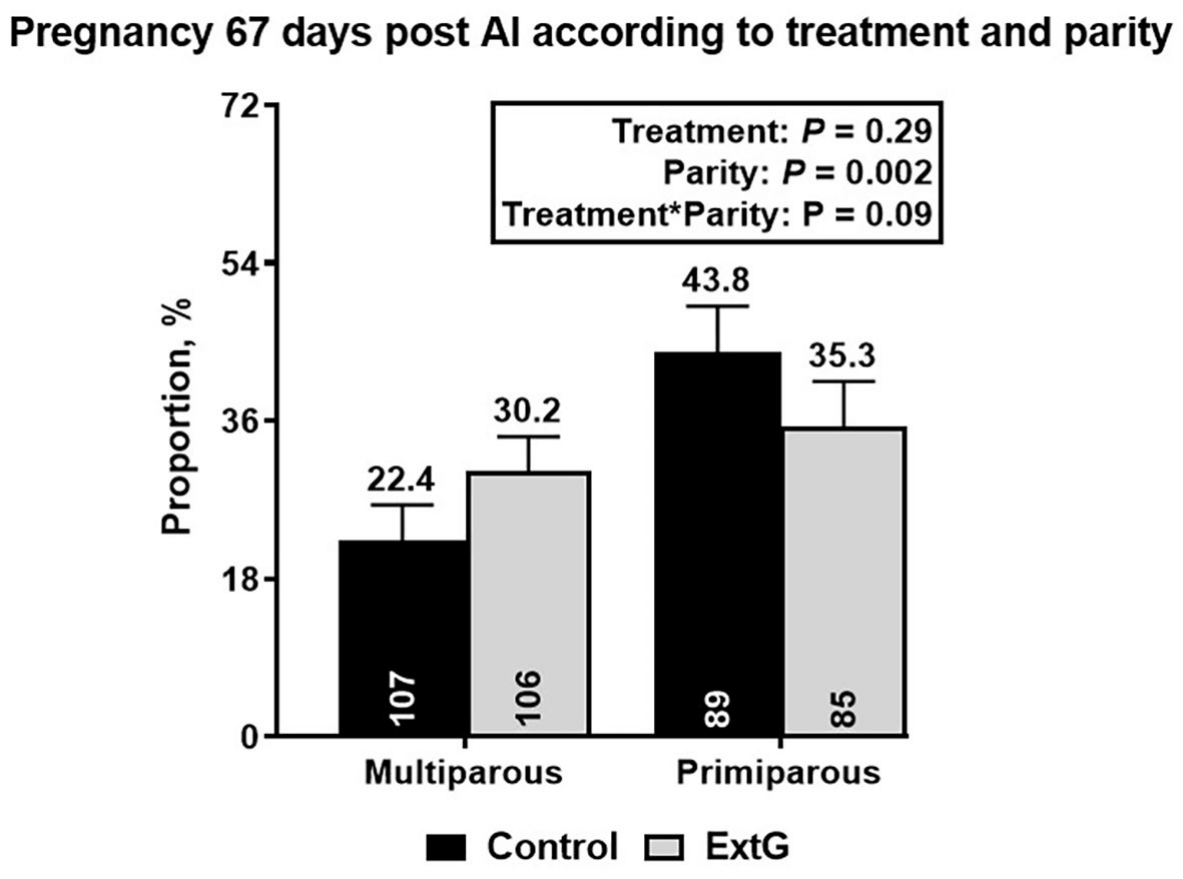
Bar graph illustrating the interaction effect between treatment and parity on pregnancy per AI at day 67 post-AI. Pregnancy per AI at day 67 post-AI was higher in primiparous cows when compared to multiparous in the Control treatment, but no differences in pregnancy per AI at day 67 post AI for ExtG were observed.

Nine cows in the ExtG treatment and ten cows in the Control treatment left the herd before receiving a pregnancy diagnosis. Thus, the final number of cows used to analyze pregnancy outcomes were 196 cows for ExtG and 191 cows for Control. Cows enrolled in the Control, and ExtG treatments had similar (*P* = 0.75) P/AI at 32 post-AI (Figure 4). There was also no difference in P/AI at days 67 post-AI (*P* = 0.29) and pregnancy loss (*P* = 0.42) between the treatments (Figure 4). There were no effects of season (cool season: November–April; warm season: May–October) on P/AI on days 32 (Cool = 33.3 ± 3.4 vs. Warm = 34.3 ±3.3, *P* = 0.95) and 67 (Cool = 32.3 ± 3.4 vs. Warm = 32.0 ± 3.3, *P* = 0.83) or for pregnancy loss (Cool = 3.2 vs. Warm = 6.2, *P* = 0.45). There was an effect of parity with primiparous cows having a greater proportion of pregnancies than multiparous cows at days 32 (*P* = 0.002) and 67 (*P* = 0.002) post AI (Figure 5). There was not an effect (*P* = 0.68) of parity on pregnancy loss (Figure 5). Moreover, there was a tendency for an interaction between treatment and parity (*P* = 0.09). In multiparous cows, ExtG tending to have greater P/AI than Control at day 67 post-AI, whereas in primiparous cows Control tended to have greater P/AI than ExtG at day 67 post-AI (Figure 6).

### Descriptive Data for Short and Long Enrollment Interval

During the experiment, the voluntary waiting period was shortened from 72 ± 3 to 65 ± 3 DIM, and consequently, the enrollment had to be adjusted accordingly from 48 ± 3 (LongEnr) to 41 ± 3 DIM (ShortEnr). Overall P/AI at day 32 (LongEnr = 36.7% vs. ShortEnr = 29.8%) and at day 67 (LongEnr = 35.0% vs. ShortEnr = 28.1%) looked numerically higher for cows enrolled during the LongEnr than during ShortEnr. Nevertheless, similar to the results observed for entire study cows in the LongEnr had no differences in P/AI at day 32 (*P* = 0.68), P/AI at day 67 post-AI (*P* = 0.67), and pregnancy loss (*P* = 0.88) between treatments (Table 3). Cows in the LongEnr had an effect of parity with primiparous having greater P/AI at days 32 (*P* = 0.02) and 67 (*P* = 0.03) post-AI than multiparous cows (Table 3). No difference between primiparous and multiparous for pregnancy loss (*P* = 0.86) was found during LongEnr. Likewise, ShortEnr cows had no differences in P/AI at day 32 (*P* = 0.18), P/AI at day 67 post AI (*P* = 0.48) and pregnancy loss (*P* = 0.17) between treatments (Table 3). Cows in the ShortEnr had an effect of parity with primiparous having greater P/AI at days 32 (*P* = 0.01) and 67 (*P* = 0.03) post-AI than multiparous cows (Table 3). No difference between primiparous and multiparous for pregnancy loss (*P* = 0.24) was found in ShortEnr (Table 3).

**Table 3:** Pregnancy per AI at day 32 post-AI, pregnancy per AI at 67 post-AI, and pregnancy loss between days 32 and 67 for cows synchronized after longer (48 ± 3) interval in days milk for enrollment (LongEnr) and shorter (41 ± 3) interval in days milk for enrollment (ShortEnr) according to treatment (Control and ExtG) and parity (Multiparous vs. Primiparous).

	Treatments^*^		**Parity**		***P*-value treatment**	***P*-value parity**
	**Control**	**ExtG**	**Multiparous**	**Primiparous**		
**Pregnancy per AI 32 days post AI**						
LongEnr (48 ±3), % (*n*/*n*)	38.3 (44/115)	35.1 (39/111)	31.0 (44/152)	46.4 (39/84)	0.68	0.02
ShortEnr (41 ±3), % (n/n)	25.9 (21/81)	33.7 (27/80)	19.7 (14/71)	37.8 (34/90)	0.18	0.01
**Pregnancy per AI 67 days post AI**						
LongEnr (48 ±3), % (*n*/*n*)	36.5 (42/115)	33.3 (37/111)	29.6 (42/142)	44.0 (37/84)	0.67	0.03
ShortEnr (41 ±3), % (*n*/*n*)	25.0 (20/80)	31.2 (25/80)	19.7 (14/71)	34.8 (32/90)	0.48	0.03
**Pregnancy loss between day 67 and 32 post-AI**						
LongEnr (48 ±3), % (*n*/*n*)	4.5 (2/44)	5.1 (2/39)	4.5 (2/44)	5.4 (2/37)	0.88	0.86
ShortEnr (41 ±3), % (*n*/*n*)	0.0 (0/20)	8.0 (2/25)	0.0 (0/14)	5.9 (2/24)	0.17	0.24

* *Cows were randomly allocated to two either Control or ExtG treatments. The control treatment consisted of presynchronization with GnRH and PGF_2α_, followed by OvSynch 7 days later, while ExtG treatment consisted of treatment of GnRH followed 7 days later by simultaneous GnRH and PGF_2α_ followed by OvSynch after 7 days*.

### Descriptive Data for an Additional Injection of PGF_2α_

During the experiment, the farm decided to add an extra PGF_2α_ to improve luteolysis. Overall P/AI at day 32 (One PGF_2α_ = 34.0% vs. Two PGF_2α_ = 33.8%) and at day 67 (One PGF_2α_ = 31.5% vs. Two PGF_2α_ = 32.6%) were very similar for cows receiving one or two PGF_2α_ treatments. Similar to the results observed for entire study cows that received only a single PGF_2α_ had no differences in P/AI at day 32 (*P* = 0.33), P/AI at day 67 post-AI (*P* = 0.33) and pregnancy loss (*P* = 0.91) between treatments (Table 4). Primiparous cows receiving a single PGF_2α_ tended (*P* = 0.07) to have a greater P/AI at days 32 post-AI (Table 4). No difference between primiparous and multiparous cows for P/AI at day 67 post-AI (*P* = 0.10) and pregnancy loss (*P* = 0.73) was found for cows receiving a single PGF_2α_ (Table 4). Cows receiving two PGF_2α_ treatments had no differences in P/AI at day 32 (*P* = 0.61), P/AI at day 67 post AI (*P* = 0.71) and pregnancy loss (*P* = 0.23) between treatments (Table 4). Cows receiving two PGF_2α_ treatments had an effect of parity with primiparous having greater P/AI at days 32 (*P* < 0.01) and 67 (*P* = 0.01) post-AI than multiparous cows (Table 4). No difference between primiparous and multiparous for pregnancy loss (*P* = 0.43) was found in cows receiving two PGF_2α_ treatments.

**Table 4:** Pregnancy per AI at day 32 post AI, pregnancy per AI at 67 post AI, and pregnancy loss between days 32 and 67 for cows receiving one or two PGF_2α_ treatment at OvSynch according to treatment (Control and ExtG) and parity (Multiparous vs. Primiparous).

	Treatments^*^		**Parity**		***P*-value treatment**	***P*-value parity**
	**Control**	**ExtG**	**Multiparous**	**Primiparous**		
**Pregnancy per AI 32 days post AI**						
One PGF_2α_ treatment, % (*n*/*n*)	31.7 (26/82)	36.2 (29/80)	29.6 (32/108)	42.6 (23/54)	0.33	0.07
Two PGF_2α_ treatment, % (*n*/*n*)	34.2 (39/114)	33.3 (37/111)	24.8 (26/105)	41.7 (50/120)	0.61	<0.01
**Pregnancy per AI 67 days post AI**						
One PGF_2α_ treatment, % (*n*/*n*)	29.3 (24/82)	33.7 (27/80)	27.8 (30/108)	44.0 (21/54)	0.33	0.10
Two PGF_2α_ treatment, % (*n*/*n*)	34.2 (39/114)	31.5 (35/111)	24.8 (26/105)	40.0 (48/120)	0.71	0.01
**Pregnancy loss between day 67 and 32 post-AI**						
One PGF_2α_ treatment, % (*n*/*n*)	7.7 (2/26)	6.9 (2/29)	6.2 (2/32)	8.7 (2/23)	0.91	0.73
Two PGF_2α_ treatment, % (*n*/*n*)	0.0 (0/39)	5.4 (2/37)	0.0 (0/26)	4.0 (2/50)	0.23	0.43

* *Cows were randomly allocated to two either Control or ExtG treatments. The control treatment consisted of presynchronization with GnRH and PGF_2α_, followed by OvSynch 7 days later, while ExtG treatment consisted of treatment of GnRH followed 7 days later by simultaneous GnRH and PGF_2α_ followed by OvSynch after 7 days*.

## Discussion

This experiment was conducted to assess whether an additional injection of GnRH 7 days before a presynchronization with PG+G would decrease the randomness of ovulatory follicles and CLs in cows receiving this program. Presumably, improved ovulatory response and CL regression after the PG+G could impact two key contributors to high pregnancy outcomes in dairy cows. First, the anticipated improved responses could increase the percentage of cows starting OvSynch in a favorable window for ovulation (days 6 to 7 of the estrous cycle), which has been shown to increase ovulation to the first GnRH and P/AI in synchronization programs (8, 12, 16, 27). Secondly, improved responses to the additional GnRH and subsequent PG+G could increase the percentage of cows with responsive CL at the start of the synchronization program, another factor critical to improving P/AI (3, 4, 28).

The anticipated improved follicular responses were limited to a tendency for increased ovulation at enrollment in ExtG cows when compared to Control cows that were left untreated. When the data were analyzed according to parity, the tendency for increased ovulation of ExtG over Control at enrollment was present in primiparous, but not in multiparous. The tendency between primiparous and multiparous might be in part explained by the fact that only 44% of primiparous cows in the ExtG had a CL, whereas 64% of the multiparous cows had a CL. A higher number of cows bearing a CL might lead to a large number of cows with high progesterone, which has been shown to reduce LH release and ovulation in cows (29) and heifers (30). Ovulation at the PG+G and the first GnRH of OvSynch were similar, not supporting our hypothesis that potentially more cows having a dominant follicle on days 6 or 7 would optimize ovulation at PG+G. Although not statistically significant, the percentage of cows ovulating to the first GnRH of the OvSynch was 8.6 percentage points higher in Control cows than in ExtG cows. The percentage of cows ovulating to the first GnRH in the current study was relatively higher (90.6 vs. 68.0%) than the previous study using PG+G presynchronization (10). It is unclear why the ovulation to the first GnRH was relatively higher in the current study than in the previous PG+G study reporting ovulation for the first GnRH. However, similar variability in ovulatory response and discrepancies in results in presynchronization have been reported for the G6G [66% (10) vs. 85% (8, 15)] and Double OvSynch [61.5% (31) vs. 80% (14)] programs. There is a multitude of factors such as environment, nutrition, history of diseases, and genetics that might help explain differences amongst studies.

The increased tendency for ovulation at enrollment for ExtG amongst primiparous cows when compared to Control cows did translate into an increased number of CL at PG+G. Albeit, when both parities were analyzed together, a 10.7 percentage point numerical increase in CL presence for ExtG cows over Control cows with a statistical significance of 0.11 was present. The increased number of CLs presence for ExtG primiparous cows over Control primiparous cows is a two-way story that can be puzzling to interpret. A higher number of cows with a CL that is responsive to PGF_2α_ (>5 days in the estrous cycle) can help improve luteolysis (19, 20). On the other hand, as discussed above, a higher number of CLs might suggest more cows with high enough progesterone to inhibit LH release and ovulation (29, 30). The presence of a CL at the first GnRH of OvSynch was very similar between treatments (Control = 75.5% vs. ExtG = 76.0%), and the previous study with PG+G that reported the presence of CL for the first GnRH of OvSynch of 76% (10). However, the current study and previous PG+G results are still lower than studies with G6G and Double-OvSynch that ranged between 85 and 91% (8–10, 14). Multiparous cows had a greater presence of CL at 1^st^ GnRH of OvSynch compared to primiparous cows. The higher percentage of cows with a CL and adequate progesterone at the beginning of OvSynch has been considered a significant contributor to the high performance of presynchronization in fertility programs (32–35). Another finding of the current study was a tendency for cows in ExtG to have large CL volume than Control cows. A larger CL volume might be suggestive of higher concentration of progesterone and older CL, which are more likely to regress after a single treatment of PGF_2α_ (36). However, progesterone and luteolysis after the second PGF_2α_ were not measured in the current study, and suggestions of potential differences are merely speculative.

Considering the parity dependent results for follicular and luteal responses for ExtG and Control in the OvSynch, it was not a surprise that differences in pregnancy outcomes were limited to the interaction between parity and treatment. Parity had effects on P/AI at days 32 and 67 post AI with primiparous cows having a greater proportion of pregnancies than multiparous cows. Higher pregnancy outcomes for primiparous over multiparous cows have been reported in several studies using or not presynchronization with GnRH (1, 9, 11, 37). In the current study, primiparous had a lower presence of CL at the first GnRH than multiparous cows. Generally, primiparous cows are more likely to be anovular than multiparous cows (1, 2, 5, 38). It has been suggested by Souza et al. (9) that a possible reason for improvements reported in primiparous cows using the Double-OvSynch was a reduction on the number of anovular cows. However, these results were not confirmed by a later study using Double-OvSynch (11) and other previous studies using different programs (5, 27, 38) and the relationship between primiparity and anovular condition remains a conundrum that needs to be further explored. In the current study, the presence of a CL at first GnRH of OvSynch in primiparous cows was lower than in multiparous cows, so it is unlikely to be a positive aspect explaining the improved P/AI.

An intriguing finding of the current study was a tendency for interaction between parity and treatment for P/AI at day 67 post-AI. For multiparous cows, ExtG tended to have greater P/AI than Control (Control = 22.4% vs. ExtG = 30.2%), whilst in primiparous cows, Control tended to have greater P/AI than ExtG (Control = 43.2% vs. ExtG = 35.3%). It is unclear what caused this opposite trend for the treatment in primiparous and multiparous cows. For primiparous cows, it seems that the increased tendency for ovulation at enrollment, followed by the increased presence of CL at PG+G, and the combination of numerically higher CL presence and lower ovulation at first GnRH of OvSynch for ExtG offer a potential explanation for the lower tendency for pregnancy at day 67 pot-AI. While for multiparous cows, the lack of clear differences for follicular and luteal responses that were anticipated for the current study makes it difficult to interpret why ExtG cows tended to have increased pregnancy per AI at day 67 post-AI and need to be further investigated.

In the current study, there were cows enrolled first at 48 ± 3 (LongEnr) and then at 41 ± 3 (ShortEnr). Also, there was a portion of cows enrolled that received an additional injection of PGF_2α_ 1 day after the first PGF_2α_ of the OvSynch to aid in complete luteolysis. Cows were not randomly assigned to these allocations, because the changes were made by the farm in response to low fertility outcomes. We performed analyses for the cows in this study for both changes to make sure that the modification did not impact the treatments differently. The same type of responses was observed for LongEnr and ShortEnr enrollment, and for one and two PGF_2α_ than the entire study groups. Cows in LongEnr and ShortEnr ended up having timed AI for the first service at 72 ± 3 and 65 ± 3, respectively. Although numerical difference suggests that going from LongEnr to ShortEnr may reduce pregnancy (LongEnr = 35.0% vs. ShortEnr = 28.1% for P/AI ay days 67 post AI), cows were not randomly assigned to LongEnr vs. ShortEnr enrollment and caution should be used interpreting these results. In the two previous studies using PG+G presynchronization for the first service, followed by OvSynch, cows received timed AI at 78 ± 3, and pregnancy outcomes range from 39% to 47%. The effect of the voluntary waiting period on pregnancy outcomes is well-established (37). However, it is essential to point out that farms using the same program and voluntary waiting period can easily have distinct pregnancy outcomes (39).

We started our experiment without the additional injection of PGF_2α_ to keep the number of days on which injections needed to be given to two. However, the advantage of using two PGF_2α_ in the OvSynch program is well-established (13, 40, 41), and a recent metanalysis ratified its benefits (42). Nonetheless, cows in either treatment were not randomly allocated to receive one or two treatments of PGF_2α_ and, therefore, cannot be used to interpret a benefit or lack of it in PG+G programs.

In conclusion, GnRH 7 days before PG+G presynchronization lead to positive and negative tendencies, respectively, in multiparous and primiparous cows for P/AI at day 67 post-AI. In primiparous cows, ExtG tended to increase ovulation and increased the proportion of cows with CL at PG+G but did not improve ovulation and CL presence at anticipated for the beginning of OvSynch. Potential benefits and detrimental responses of adding GnRH 7 days before PG+G in primiparous and multiparous cows need further investigation.

## Data Availability Statement

The raw data supporting the conclusions of this article will be made available by the authors, without undue reservation.

## Ethics Statement

Ethical review and approval was not required for the animal study because this study was part of routine farm work. However, all animal procedures followed the recommendations of the Guide for the Care and Use of Agricultural Animals in Agricultural Research and Teaching (FASS, 2010; accessed at https://www.adsa.org/Portals/_default/SiteContent/docs/AgGuide3rd/Chapter07.pdf). Written informed consent for participation was not obtained from the owners because the owner was a long time client of the first author of the manuscript, AH, and provided consent to conduct the study.

## Author Contributions

AH: conceptualization, investigation, formal analysis, data curation, writing—original draft, writing—review, and editing. PP and JH: investigation. IC: formal analysis, data curation, writing—review, and editing. FL: conceptualization, investigation, formal analysis, data curation, funding acquisition, writing—review, and editing. All authors contributed to the article and approved the submitted version.

## Conflict of Interest

The authors declare that the research was conducted in the absence of any commercial or financial relationships that could be construed as a potential conflict of interest.
